# The *Time Moving* exhibit: Exploring perceptions of time in end‐of‐life experiences

**DOI:** 10.1111/hex.13379

**Published:** 2022-01-15

**Authors:** Kate Sellen, Molly McGovern, Emma MacGregor, Laura Halleran, Lawrence Ly

**Affiliations:** ^1^ Health Design Studio OCAD University Toronto Ontario Canada

**Keywords:** art, decision‐making, design research, end‐of‐life, human‐centred design

## Abstract

**Background:**

Conversations about end of life rarely take place beyond healthcare spaces and in advance of imminent death. As the Canadian ageing population increases and new policies and options emerge for end‐of‐life choices, there will be an increasing need for supports for decision‐making on end of life. *Time Moving* was a participatory art exhibit created to engage participants in reflections on end‐of‐life experiences and the ways in which their perception of time fluctuated within these moments.

**Design:**

A participatory and constructivist approach informed decisions on exhibit design and analysis. The *Time Moving* exhibit was installed in a downtown university hall for 10 days during the 2019 annual DesignTO festival, open to all members of the public. The exhibit was used as the primary method of data collection. There were three prompts informed by concepts from prior work on time perception. Participants were invited to respond by drawing, writing or constructing.

**Outcome:**

There were over 120 contributions of participants over the duration of the Time Moving exhibit. These contributions revealed new insights related to temporal perception and end‐of‐life experiences. Participants shared their perception of time during end‐of‐life experiences in a variety of ways, revealing a diversity of perceptions of time beyond calendar and clock time, including time experienced as a spiral.

**Conclusions:**

The insights on time perception highlight opportunities for approaching dialogue on end of life and in grief support, and for exhibit as a mechanism for research and education.

**Patient or Public Contribution:**

As a public exhibit, over 120 people contributed ideas, personal stories, drawings and other creative outputs to the exhibit. An estimated 250 people visited the exhibit.

## 1 INTRODUCTION

Like other nations, Canada is on the cusp of experiencing a peak population. Baby boomers are ageing, signalling that the number of people aged 65 years and older will increase dramatically over the next 20–30 years.^1^ As Canada's population ages, the demand for end‐of‐life care will grow. It is estimated that by 2031, about 350,000 people will die each year in Canada—up from 250,000 in 2001.^1^ The research indicates that coordination of responsibilities and tasks that support all aspects of a dying loved one's needs is a major concern for families and unequal distribution of tasks can be a source of interpersonal conflict.^2^, ^3^ With a sensitive topic like death and dying, a common issue is knowing how to start conversations on planning, needs and wishes.^4^ Conversations about experiences with dying have very few places in which they can occur commonly and without judgement.^5^ Despite this barrier, it is widely accepted that meaningful reflections on death and dying before the end‐of‐life process begins are essential for supportive decision‐making^6^, ^7^, ^8^ including advanced care planning. In end‐of‐life circumstances, perception of time can change as temporal awareness shifts, priorities change, illnesses advance and capacity and physical constraints arise; this in turn can make decision‐making harder.

The literature on participation in decision‐making at end of life suggests that three areas need to be in place for effective participation in everyday (personal hygiene decisions) and longer‐term decisions: information sharing with the patient; accepting prognosis; spirituality; and sense of well‐being.^9^ Under emotional stress (absence of sense of well‐being), patients can perceive a slowing of time, overestimating the duration of an event,^10^ again complicating decision‐making. End‐of‐life decision‐making for some patients is further complicated by an orientation to recurring events or memories as markers of time.^11^ This is also seen with palliative cancer patients in pain and dementia patients,^12^ where memory events (dementia) or pain events (cancer) dominate time perception and can be experienced as cyclical. Decision‐making in these circumstances might then involve revisiting options and decisions.^13^ Research on decision‐making and brain function further indicates that modulations in time perspectives and dynamic relationships within subjective time perception determine time‐related decision‐making and behavioural choices.^14^

Time perception specifically related to end‐of‐life experiences and care decisions is under‐researched.^15^ The *Time Moving* exhibit comes at a point in time when some are questioning the limits of traditional biomedical approaches to death and dying,^16^, ^17^ design‐oriented projects and studios are altering conceptions of palliative space,^18^, ^19^ game designers are creating innovative ways to open up understandings and conversations about death^20^, ^21^, ^22^, ^23^ and practitioners, activists and researchers are working to understand the impact of class, race and ethnicity on the experience of death and dying.^24^

Existing research on time perception intended to inform palliative and end‐of‐life care is sparse. Exceptions include Glaser and Straus's work, which dates from the 1960s.^25^ The intent of most of the research addressing time perception and end of life is to support those experiencing a life‐limiting illness and those who are at or heading towards the end of life.^12^, ^26^ Rasmussen and Elverdam^15^ explored perception of time in cancer survivors, highlighting the disruption of clock time and calendar time, and an increased reflection on and heightened awareness of time. Martino and Freda^26^ provide a detailed review of existing psychological research on time, which highlighted the predominance of work on those experiencing chronic illness and/or decline as opposed to those witnessing decline and death, or those who are family/caregivers and involved in decision‐making or carrying out decisions. Pestinger et al.^27^ studied the motivations for palliative patients to request a hastened death and found that self‐determination, agony and time were main themes. Time perception, in this regard, relates to the experience of time periods of pain, waiting and knowing that time is limited. Rovers et al.'s^12^ work on palliative cancer patients' time perception highlights the perception of time as shifting (pace specifically), and the dominance of past and future time orientations (separately). Directly quoting from this study, participants reported: ‘the future is a big circle, because I do not know what the future holds’. ‘The future used to be much more important, I was a planner’. Research with cancer survivors revealed that when looking back on the crisis of diagnosis and treatment, it is as if in a ‘state of paralyzed memory, overwhelmed by an experience that is absorbing all possible developments like a black hole’.^26^ Given a contrast between noncrisis periods of life when time passes as calendar and clock time and where more generally a future orientation enables planning and decision‐making,^28^ the research on time perception at end of life indicates a challenge in decision‐making.^29^ This manifests as living day to day, unable to make big decisions or to engage in larger care decisions.^10^

### 1.1 Aims

As part of a broader research programme on time and communication and decision‐making during the end of life, this study, *Time Moving*, aims to explore perception of time in end‐of‐life experiences to inform supportive tools and initiatives for end‐of‐life conversations and decision‐making.

## 2 METHODOLOGY

Time Moving builds upon arts‐informed health research approaches,^30^, ^31^ and is theoretically situated in the tradition of participatory design and constructivism,^32^ drawing on the prior work of artists and designers working on death and dying.^33^, ^34^, ^35^ The exhibit took 3 months to design and is informed by the work of Glaser and Strauss,^36^ as well as concepts from health philosophy including emotional oscillation,^37^ liminal states^38^ and temporal frames.^39^ Additional details of the design of the exhibit are described elsewhere^40^, ^41^, ^42^:

1. People have different ways they think about time. Choose or draw a shape that looks the most like time to you.
2. There are many ways we experience time in end of life; what did time feel like in your experience?
3. How is dying time different from living time? What does time look like? Share a story in yarn, shapes and so forth.

**Figure 1:**
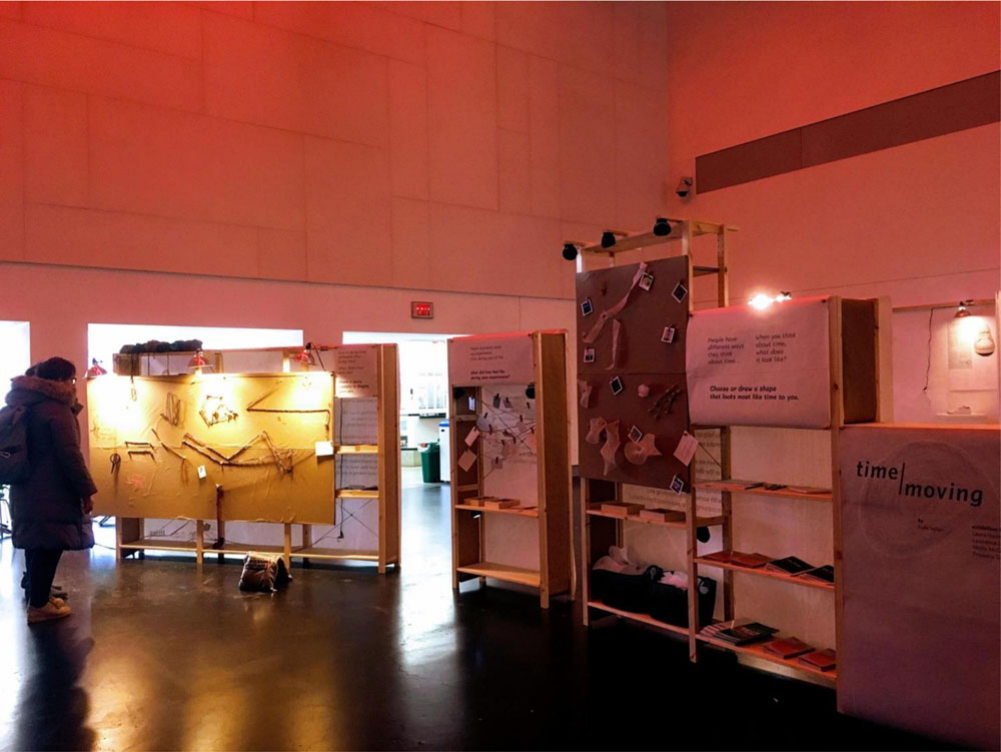
*Time Moving* installed as part of the Dying. series at DesignTO

Following participatory practice and use of materials,^43^ members of the public were invited to use a variety of everyday materials (chalk, yarn, rubber bands, charcoal, etc.) to respond to these prompts (see Figure 1). The format and shape of the exhibit were designed to provide a sense of being in a separate but open space. The use of semi‐transparent paper on the exterior of the exhibit structure, draped and loosely pinned to the structure, echoes the concept of time as fluid.

If the public had questions about their participation, facilitators were available in person at the site during exhibit hours, as well as during scheduled times. In the event that the exhibit triggered difficult emotions or sensations, postcards with supportive mental health resources and local organisations (Death Cafe,^44^ Death Over Dinner^45^) were openly available at the site and facilitators were on hand to direct participants to these resources. Facilitators otherwise did not prompt or interact with participants. The researcher's email was included on this postcard, for participants to ask further questions, request a copy of the results or to inquire into future research activities. This study received REB approval from the OCAD University institutional review board: approval #101472.

### 2.1 Setting

Time Moving was exhibited as part of an annual death event series in Toronto, Canada. The Dying. team organized a diverse set of events and activities designed to bring dying and death into conversation. The Dying. event series was a part of the larger city‐wide design festival: DesignTO.^41^ Time Moving was mounted in the main hall of OCAD University, Toronto, for 10 days (the duration of the DesignTO festival).

### 2.2 Data collection

Data were contributed by members of the public throughout the festival. The responses were then carefully secured (on the large board) and collected at the end of the exhibition. All responses were then recorded by the research team, including all markings on the installation.

### 2.3 Data analysis

**Figure 2:**
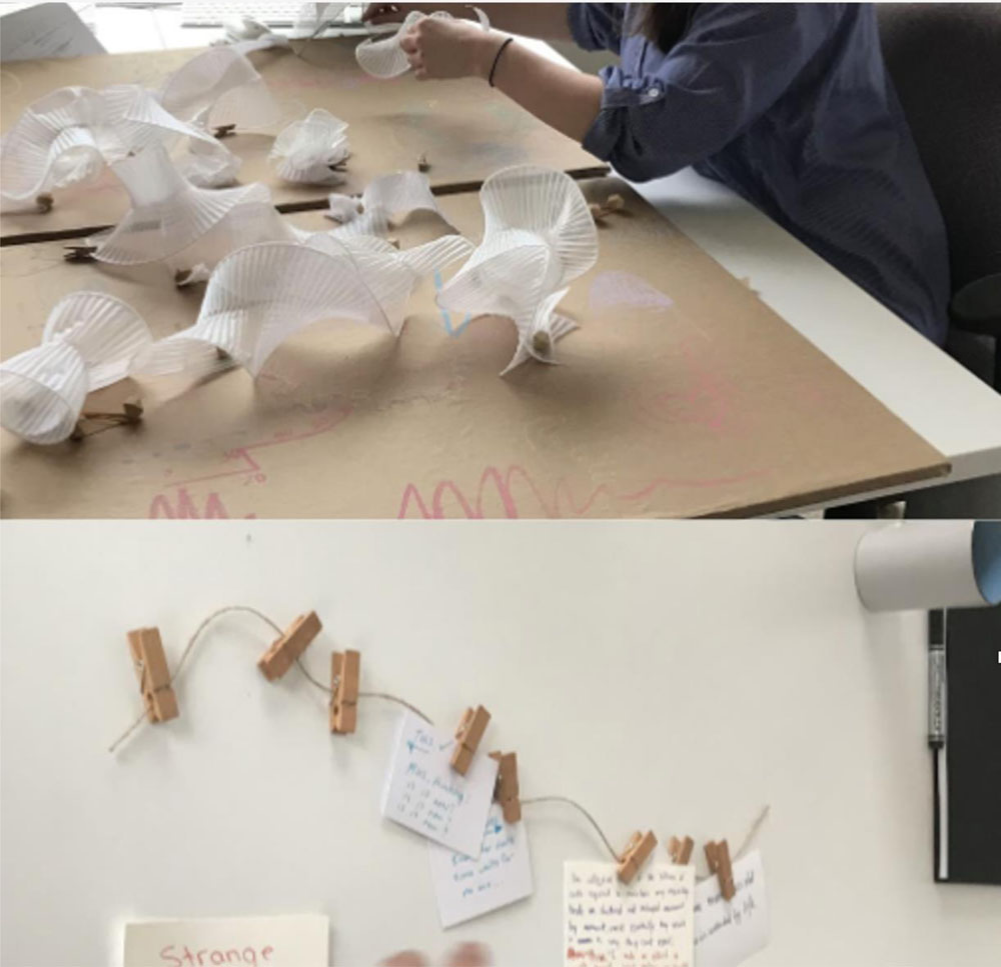
Process photos from the analysis phase

To analyse *Time Moving*, we used an inductive qualitative approach for text‐based contributions^46^ and visual analysis techniques for visual contributions.^47^ Multiple researchers were involved in every stage of the analysis process (Figure 2), and all researchers had prior training in qualitative analysis in health‐based research. Data were first photographed; each postcard was then also photographed and given a code that identified location or relationship to adjacent contributions. The first phase of analysis consisted of physical sorting and resorting of the postcards by two researchers. In the case where participants had written a response to the prompts (pinned postcard or written on paper), the responses were sorted into common groupings, with four researchers reviewing and refining. Visual drawings and material constructions were provisionally grouped by shape. A second round of review and reflection for each type of data was undertaken with researchers switching data type. At this stage, visual patterns were redrawn into a common visual format (black ink on white card). This process enabled foregrounding of the shape of participants' perceptions of time. Following this step, all researchers reviewed the provisional groupings and contributed to interpretation and descriptions of these groupings. Three researchers then separately wrote a description of the largest panel, rewriting and reviewing each of these into one descriptive interpretation. It was during this process that the term tapestry emerged as an appropriate description for the interwoven nature of participants' many contributions to the third panel.

## 3 RESULTS

Over 250 individuals visited the exhibit, with approximately 120 contributions made by visitors/participants. It is not possible to estimate the exact number of individuals participating. Participants were not required to provide details of their identities, so the demographics of participation in Time Moving remain unknown. Over 120 contributions were analysed in the data analysis phase of the project.

Participants contributed a range of responses from members of the public with representations of temporal patterns during end‐of‐life experiences. The result is a tapestry of narratives and reflections on end‐of‐life experiences, orientation to time horizons and perceptions of time woven together. The result highlights curiosity and reflection on the unfolding of events, decisions made and experiences with end of life.

### 3.1 What does time look like?

**Figure 3:**
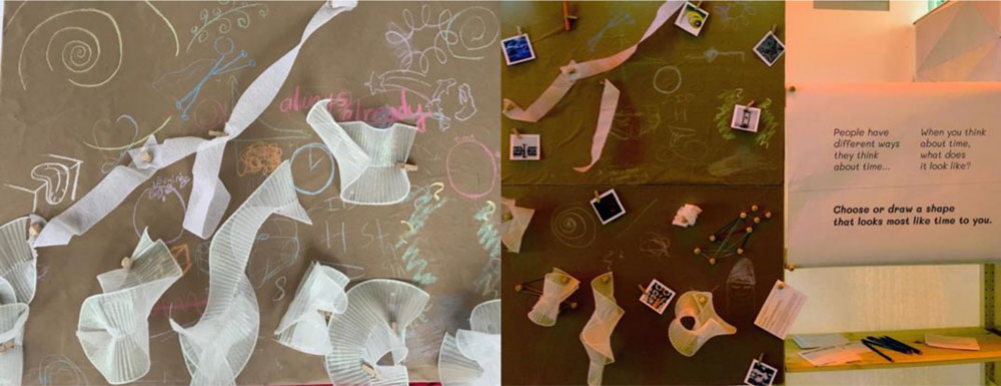
Panel one of *Time Moving*

The first panel of *Time Moving* (Figure 3) asked participants to respond to the following prompt: *People have different ways they think about time. Choose or draw a shape that looks the most like time to you*. In reviewing the responses, we found that participants chose primarily to draw but also to add descriptive adjectives. We identified various categories of time that were used by participants to describe their temporal perception in relation to end of life (the topic of the exhibit). We identified four categories: (1) unsettling, (2) supportive, (3) measurements and (4) complex.

(1) Unsettling descriptors and images suggest that there was not enough time and participants felt unsettled from this experience. Participants wrote words like ‘disoriented’. (2) Supportive adjectives like ‘blissful’ and drawings of peaceful images depicting people looking content suggest that time felt stable. (3) Participants used adjectives to describe how time felt, such as ‘stretchy’ and ‘slow’, with drawings of lines to show how time felt during their experiences of death and dying. (4) Complex adjectives were also included—‘surreal’, ‘mishmashed’ and ‘strange’ and spiral images suggested that for some, time felt unconventional and was outside of ways participants may feel or experience time in other significant moments of their lives.

### 3.2 What does time feel like?

**Figure 4:**
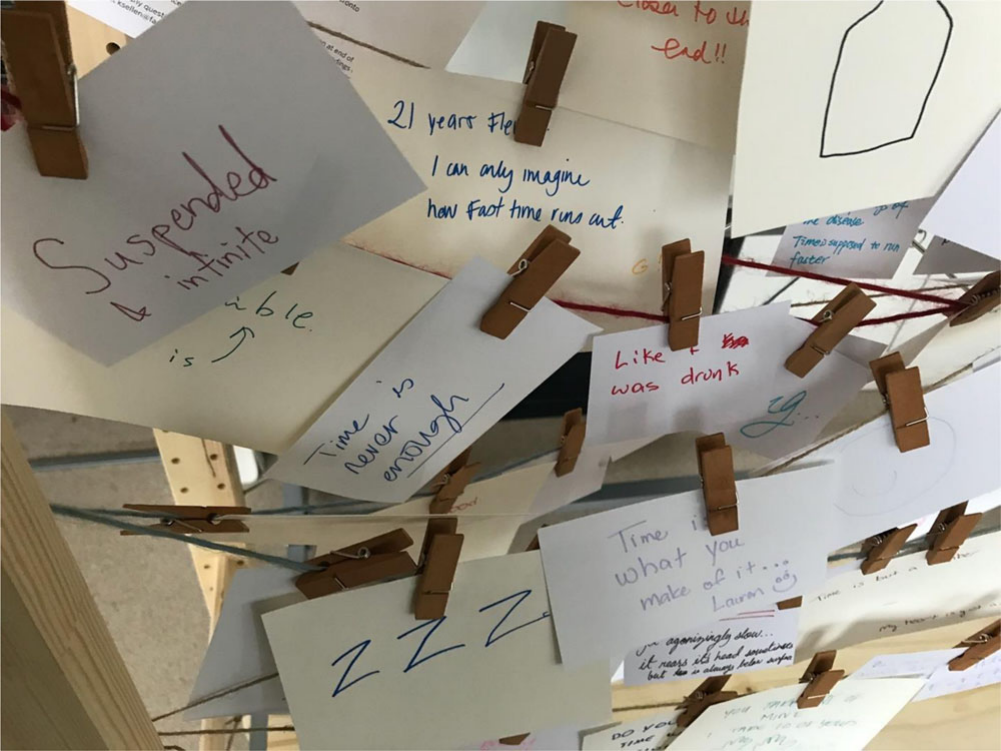
Cards strung up in Panel 2

*Time Moving* asked participants to respond to the following prompt: *There are many ways we experience time in the end of life, what did time feel like in your experience?* (Figure 4). In reviewing the responses, we found that participants began to use metaphors/similes to explore how the speed of time changed in end of life. For example, one participant wrote ‘like it was more real and going too fast’. ‘Time is like a bubble we sit inside and wait to pop’. Other answers explored how time in the presence of death felt long and unending. One participant wrote ‘Like sipping it non‐stop into my body but I couldn't save any of it’, suggesting that time in the end of life moved too fast and a sense of loss of control over the experience. ‘The most objective unbiased serial killer of everything’. Others used simile to help explain the range of emotions that they felt in their experience with death. ‘Like playing a role no one prepared you for. Periods of intense waiting. Moments you don't want to end, for the joy and the sorrow, an inability to face what's next’. These examples illustrate how time was felt as an entity as something in liquid or gas form, something tangible or even alive (serial killer), enveloping, consumable, but separate. Participants proposed that time was objective, and that death was an unavoidable repercussion of time. Relating to this, their daily life comments suggested accepting no control over time and focus on positive emotions during the life course.

‘live life like a traveler leaving permanent footprints in a temporary world’

‘if I can't stand my own ground how can I find my way out of this maze?’

### 3.3 Dying time

**Figure 5:**
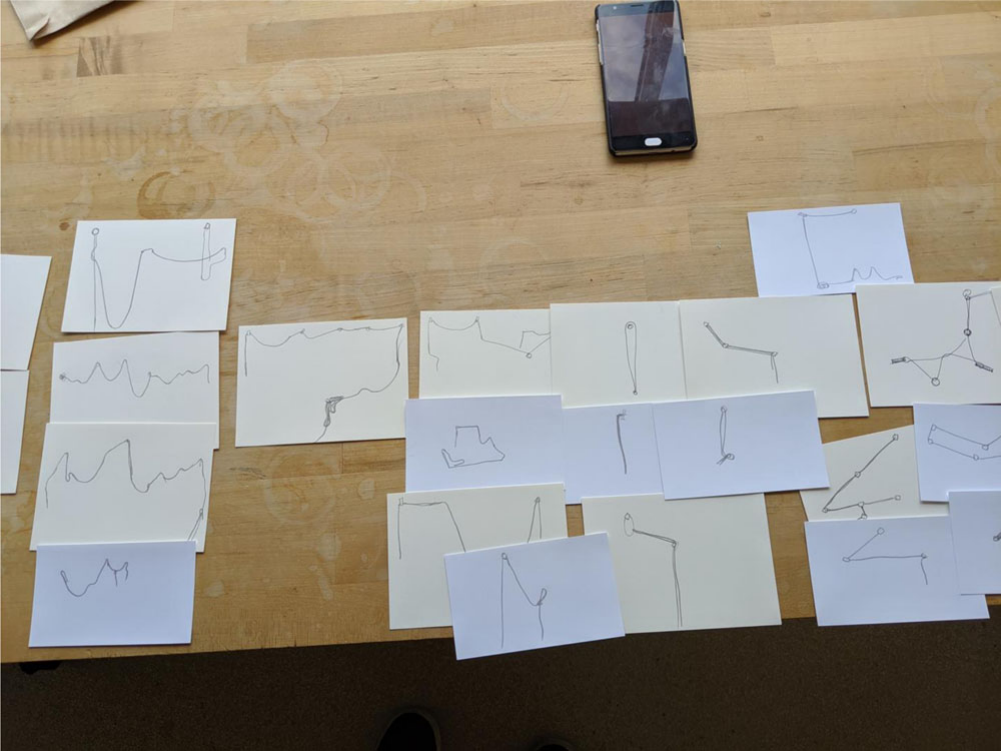
Process of visual analysis included redrawing string forms on small cards

The third panel of *Time Moving* asked participants to respond to the following prompt: *How is dying time different from living time? What does time look like? Share a story in yarn, shapes and so forth*. Analysis (Figure 5) indicated three categories: (1) loose linear and chaotic timelines, (2) structured open and closed conceptualisations of time and (3) highly structured conceptualisations.

**Figure 6:**
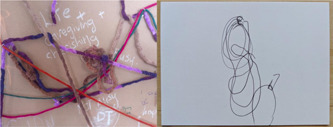
Loose linear shape on tapestry, and sketch example

**Figure 7:**
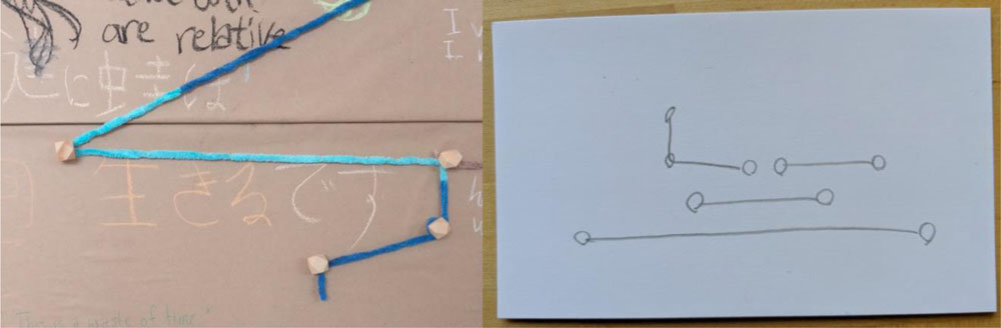
Structured‐open shape on tapestry, and sketch example

**Figure 8:**
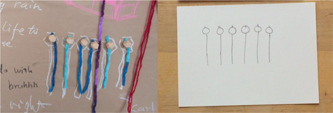
Highly structured shape on tapestry, and sketch example.

(1) Loose linear and chaotic timelines. The structures in this category are hanging loosely, but begin to take on a linear form at some point in the shape. The forms remain abstract and organic as the shapes made with the materials are free flowing and lack a defined geometry (Figure 6).

(2) Structured open and closed conceptualisations of time (Figure 7). In this category, the shapes are taut and hold a linear pattern that is very defined, but the structure remains open, perhaps following more closely than other contributions to a linear conceptualisation of time connected to calendar and clock time. The materials form a straight line, or a collection of straight lines strung together. Additionally, we also found shapes that were geometric, taut and held a defined linear pattern where the shape has been closed by the participant.

(3) Highly structured conceptualisations (Figure 8). In this category, the shapes were identified that were intentional in their representation of traditionally understood shapes (i.e., triangles, hearts) or symbols.

### 3.4 Time Moving as a whole

**Figure 9:**
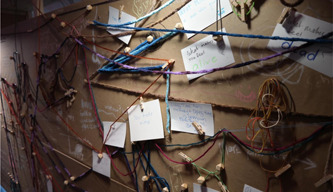
*Time Moving* panel after the exhibition was finished

Time Moving collectively can be viewed as a tapestry, threads hanging and woven together into meaningful groupings and connections, made up of individual vignettes, but with a common theme (Figure 9). The piece opens gently with small abstract drawings and phrases exploring the dying experience, before leading in a complex middle with yarn, rubber bands and graph drawings. The middle section of the piece is complex, dense and messy. Yarn and other materials go over each other, with no configuration of one dying experience emerging. The end of *Time Moving* leads to a collection of index cards sharing personal experiences and wisdoms about death and dying.

The contributions to *Time Moving* ranged from identifiable depictions of concrete objects and symbols, open‐ended structures, linear and loose depictions of lines or journeys, to abstract representations. Numerous contributions displayed a conventional approach to timelines, using chalk or yarn to draw linear paths across the tapestry. Others included annotations to describe the reasoning behind the path directions, which helped to clarify the participant's intention and provide insight into the participant's experience with dying and time including meaningful points such as diagnosis, treatment and points of decline. As the responses grew throughout the exhibition, a strong theme of dying time as separate from everyday living time emerged. Participants questioned if death and life were the same thing and how time fit within their perceptions of these experiences. When they explored how time was different in moments of death and dying, many expanded on how dying time was unknown. Living time was described using active words like ‘awake’, ‘gaining memories’ and dying time with more passive words to describe this experience: ‘long sleep’, ‘finite’ and ‘final’. Participants shared reflections of their experiences with time, expressing that time felt both fast/sudden and long/slow. Participants suggested that time did not present itself uniformly; it was multifaceted and unpredictable. Overall, the tapestry became a broader reflection on death and dying.

## 4 DISCUSSION

Interactive exhibits create a public presence for end‐of‐life conversations outside of traditional death experiences (wakes, funerals, celebrations of life, religious ceremony).^31^ They encourage the viewers to express their point of view in how they interact with the materials and content of the work. In the case of *Time Moving*, this interactive exhibit contributes insights on perception of time and health practice, health research and education.

### 4.1 Perception of time and end of life

*Time Moving* comprised short, discrete visual and text‐based narratives that, in their brevity, expressed the complex experience of death and dying. Each panel of *Time Moving* was filled with drawings of timelines, representing narrative arcs in many cases, but not all, with participants highlighting the ways in which dying had created disorienting moments within their own lives. Many drawings are turbulent, with ups and downs in between each key moment indicated. The tapestry of *Time Moving* speaks to a collective narrative made up of smaller vignettes encapsulating the unease around endings and the challenge of trying to make sense of time as death unfolds. Participants expressed how overwhelming time can feel and their unease with the lack of individual control involved when thinking about time. In reflecting on these categories further, the data indicate that time is experienced in a diversity of ways and is depicted in ways that support prior research indicating that time perception may not follow calendar or clock time during periods of stressful or traumatic circumstances.^48^ Given how dominant depictions of calendar and clock time are, its notable that participants, for the most part, did not draw clocks or calendar‐style representations. In Time Moving, we also found the spiral, closed and event‐based expressions of time identified in qualitative inquiry into time perception at end of life.

### 4.2 Perception of time, end of life and supportive practice

Time Moving fits into a wider movement (nurses, social workers, grief counsellors, etc.) towards supportive practices that embrace death as a natural part of life, wherein time perception shift is accepted and supported as a normal part of this experience. Time Moving may serve to further normalize the experience of time perception shifting when encountering death and dying. Unlike rational approaches to conceptualizing the dying process (such as the palliative performance scale)^49^ that may be used to trigger conversations on decision‐making and options, individual end‐of‐life trajectories depicted by the public participants of Time Moving provide alternative conceptualisation of the dying process that may facilitate a different approach to supportive and relatable conversations on time and choices at end of life. If conversations on decision‐making foreground the likelihood of decline and death being a nonlinear experience, this may encourage focus on beliefs, values and shared decision‐making earlier rather than later.

Time Moving also has relevance to practice theory in healthcare and the use of participatory techniques. Person‐centred care^50^ is a widely recognized mid‐range practice nursing theory. The theory structures prerequisites, environments and processes of person‐centred care. These components, when working in harmony, create outcomes including satisfaction and involvement with care, feeling of well‐being and creation of a therapeutic culture. Despite person‐centred care being highly recognized in the healthcare literature, it is an underexplored concept in health design scholarship. Wolstenholme et al.^51^ have discussed the role that participatory design can play in supporting the processes of person‐centred care. Having conducted a participatory design session with nurses and older adults, analysis found that the participants expressed sentiments that matched with a majority of four tenets of McCormack and McCance's person‐centred care.^52^ These tenets are knowing self, engagement, working with a patient's beliefs and values and shared decision‐making and involvement in care.^9^ We suggest that Time Moving also presents an opportunity to encourage conversation on person‐centred care.

### 4.3 Participatory exhibit as education

In healthcare education, exhibits can be used to create new experiential learning opportunities and support interdisciplinary education on a wide range of health perspectives.^27^ The installation encourages cocreation of knowledge and dialogue, which can be useful to support interdisciplinary dialogue in healthcare. Providing education on death and dying requires precious time and space in the healthcare curriculum. Such curriculum‐focused interventions may focus on concepts and frameworks of death and dying, such as the Stages of Grief,^53^ which may serve to support a theoretical understanding of the concepts. While not a specific aim of this study, *Time Moving* may serve to support educational objectives for end‐of‐life care training. By engaging in *Time Moving*, participants were able to reflect on experience, a key tenet of experiential learning.^54^ The knowledge coproduced within *Time Moving* suggests the depth of the design opportunities within this context, and the potential for the use of exhibit applied to other health education topics.

### 4.4 Participatory exhibit as research

The use of exhibit as a mechanism for data gathering for time perception research is a novel approach and one that yielded a large amount of qualitative data. Using traditional qualitative methodologies when exploring sensitive subject matter can be challenging for both the researcher and the participants. Researchers may face challenges in remaining objective, and not being swayed by emotional recounting(s) from participants.^55^ Participants themselves may face emotional stress in sharing information from these personal experiences.^55^ Open participatory exhibits offer participants a unique opportunity to engage in a dialogue about sensitive topics, like death and dying.^56^ Participatory exhibitions are an anonymous, invitational way to enable public participation in research.^57^ Participatory exhibitions can further be a creative way for researchers to engage the public in knowledge mobilisation.^42^, ^58^ Researchers can use exhibits to embody and present knowledge from established research and theory to the general public.^54^ How the public then engages with the materials within the exhibit has the potential to produce new knowledge and creates a space in which the public can explore and ask questions about complex problems. The exhibit itself becomes an artefact of this knowledge development process.^57^ In this case, Time Moving became more than a novel device for data collection. It became a tapestry of knowledge, insights and directions for future exploration that remains intact for future exhibits. Time Moving builds on exhibit and arts‐based health research^31^ and a growing interest in exhibit as a mechanism for both research^56^ and knowledge mobilisation.^58^

## 5 CONCLUSION

Death and dying is a sensitive topic that is often avoided in everyday conversation.^4^ To open these conversations, we designed *Time Moving*, a participatory exhibit that probed into the public's experiences with death and how these experiences related to the perception of time. The outcome of this study was a tapestry of responses that illustrate a diversity of perception of time and of death and dying. The contributions of participants may appear chaotic, ambiguous and overwhelming, mirroring the experience of end of life. Exploring and acknowledging the disorientation that comes with end‐of‐life experiences, this study contributes examples of perceptions of time useful for practitioners facilitating end‐of‐life conversations and communicating with family members. The work also provides an example of exhibits as research and as a potential educational tool.

## CONFLICT OF INTERESTS

The authors declare that there are no conflict of interests.

## AUTHOR CONTRIBUTIONS

Kate Sellen, Molly McGovern, Laura Halleran and Lawrence Ly have made substantial contributions to conception and design, or acquisition of data or analysis and interpretation of data. Kate Sellen, Molly McGovern and Laura Halleran were involved in drafting the manuscript or revising it critically for important intellectual content. Kate Sellen, Molly McGovern, Laura Halleran and Lawrence Ly gave final approval of the version to be published. Each author should have participated sufficiently in the work to take public responsibility for appropriate portions of the content. Kate Sellen, Molly McGovern, Laura Halleran and Lawrence Ly agreed to be accountable for all aspects of the work in ensuring that questions related to the accuracy or integrity of any part of the work are appropriately investigated and resolved.

## Data Availability

The data that support the findings of this study are available on request from the corresponding author. The data are not publicly available due to privacy or ethical restrictions.

## References

[1] Bohnert N , Chagnon J , Coulombe S , Dion P , Martel L . *Population projections for Canada (2013 to 2063), provinces and territories (2013 to 2038): technical report on methodology and assumptions*. Statistics Canada; 2015. Accessed December 16, 2020. https://www150.statcan.gc.ca/n1/en/pub/91-620-x/91-620-x2014001-eng.pdf?st=MAtlCh0u

[2] Mazanec P , Daly BJ , Ferrell BR , Prince‐Paul M . Lack of communication and control: experiences of distance caregivers of parents with advanced cancer [published correction appears in Oncol Nurs Forum. 2011 Nov;38(6):617]. Oncol Nurs Forum. 2011;38(3):307‐313. 10.1188/11.ONF.307-313 21531681 PMC3164591

[3] Molloy DW , Clarnette RM , Braun EA , Eisemann MR , Sneiderman B . Decision making in the incompetent elderly: “The Daughter from California syndrome”. J Am Geriatr Soc. 1991;39(4):396‐399. 10.1111/j.1532-5415.1991.tb02907.x 2010590

[4] Van Scoy LJ , Green MJ , Reading JM , Scott AM , Chuang CH , Levi BH . Can playing an end‐of‐life conversation game motivate people to engage in advance care planning? Am J Hosp Palliat Care. 2017 Sep;34(8):754‐761. 10.1177/1049909116656353 27406696 PMC6055477

[5] Wildfeuer J , Schnell MW , Schulz C . Talking about dying and death: on new discursive constructions of a formerly postulated taboo. Discourse Soc. 2015;26(3):366‐390. 10.1177/0957926514564739

[6] Brinkman‐Stoppelenburg A , Rietjens JA , van der Heide A . The effects of advance care planning on end‐of‐life care: a systematic review. Palliat Med. 2014;28(8):1000‐1025. 10.1177/0269216314526272 24651708

[7] Simon J , Porterfield P , Bouchal SR , Heyland D . ‘Not yet’ and ‘Just ask’: barriers and facilitators to advance care planning—a qualitative descriptive study of the perspectives of seriously ill, older patients and their families. BMJ Support Palliat Care. 2015;5(1):54‐62. 10.1136/bmjspcare-2013-000487 24644192

[8] Drentea P , Williams BR , Bailey FA , Burgio KL . “He's on his dying bed”: next‐of‐kin's experiences of the dying body. Death Stud. 2016;40(1):1‐10. 10.1080/07481187.2015.1056565 26086748

[9] Löckenhoff CE , Rutt JL . Age differences in time perception and their implications for decision making across the life span. Aging Decis Making. 2015:213‐233. 10.1016/b978-0-12-417148-0.00011-x

[10] Weissman DE . Decision making at a time of crisis near the end of life. JAMA. 2004;292(14):1738‐1743. 10.1001/jama.292.14.1738 15479939

[11] Bruss FT , Rüschendorf L . On the perception of time. Gerontology. 2010;56(4):361‐370. 10.1159/000272315 20051665

[12] Rovers JJ , Knol EJ , Pieksma J , Nienhuis W , Wichmann AB , Engels Y . Living at the end‐of‐life: experience of time of patients with cancer. BMC Palliat Care. 2019;18(1):1‐8. 31088442 10.1186/s12904-019-0424-7 PMC6518794

[13] Wenrich MD , Curtis JR , Shannon SE , Carline JD , Ambrozy DM , Ramsey PG . Communicating with dying patients within the spectrum of medical care from terminal diagnosis to death. Arch Intern Med. 2001;161(6):868‐874. 10.1001/archinte.161.6.868 11268231

[14] Wittmann M , Paulus MP . How the experience of time shapes decision‐making. Neuroeconomics. 2016:133‐144. 10.1007/978-3-642-35923-1_8

[15] Rasmussen DM , Elverdam B . Cancer survivors' experience of time: time disruption and time appropriation. J Adv Nurs. 2007;57(6):614‐622. 10.1111/j.1365-2648.2006.04133.x 17346320

[16] Gawande A . Being Mortal: Medicine and What Matters in the End. Metropolitan Books; 2014.

[17] Miller BJ . What really matters at the End of Life (videorecording); 2015.

[18] About Moth . Moth. Accessed April 20, 2021. https://moth.org.uk/ABOUT

[19] Redesigning Death: Helping people live fully right up to the end. IDEO. https://www.ideo.com/case-study/redesigning-death. Accessed April 20, 2021.

[20] That Dragon Cancer . Numinous Games. Accessed April 20, 2021. http://www.thatdragoncancer.com/#home

[21] Josef Fares & Starbreeze Studios . Brothers a Tale of Two Sons [computer game]. 505 Games. 2013.

[22] Tale of Tales. The Graveyard [computer game]. Valve Corporation. 2009.

[23] Van Scoy LJ . Hello [board game]. Common Practice. 2017.

[24] Sumalinog R , Harrington K , Dosani N , Hwang SW . Advance care planning, palliative care, and end‐of‐life care interventions for homeless people: a systematic review. Palliat Med. 2017;31(2):109‐119. 27260169 10.1177/0269216316649334

[25] Glaser B , Strauss A . Temporal aspects of dying as a non‐scheduled status passage. AJS. 1965;71:48‐59. 14336667 10.1086/223992

[26] Martino ML , Freda MF . Meaning‐making process related to temporality during breast cancer traumatic experience: the clinical use of narrative to promote a new continuity of life. Eur J Psychol. 2016;12(4):622‐634. 10.5964/ejop.v12i4.1150 27872670 PMC5114876

[27] Pestinger M , Stiel S , Elsner F , et al. The desire to hasten death: using Grounded Theory for a better understanding “When perception of time tends to be a slippery slope”. Palliat Med. 2015;29(8):711‐719. 10.1177/0269216315577748 25802321

[28] Keinan G , Friedland N , Ben‐Porath Y . Decision making under stress: scanning of alternatives under physical threat. Acta Psychol. 1987;64(3):219‐228. 10.1016/0001-6918(87)90008-4 3572731

[29] Zivkovic T . Forecasting and foreclosing futures: the temporal dissonance of advance care directives. Soc Sci Med. 2018;215:16‐22. 10.1016/j.socscimed.2018.08.035 30196148

[30] Chamberlain P , Yoxall A . ‘Of Mice and Men’: the role of interactive exhibitions as research tools for inclusive design. Des J. 2012;15(1):57‐78.

[31] Eaves S . From art for arts sake to art as means of knowing:a rationale for advancing arts‐based methods in research, practice and pedagogy. Electron J Bus Res Methods. 2014;12(2):147‐159.

[32] Spinuzzi C . The methodology of participatory design. Techn Commun. 2005;52:163‐174.

[33] Chang C . Before I die I want to. TED video. 2012;6:20.

[34] Concannon K . Yoko Ono's Dreams: the power of positive wishing. Perform Res. 2014;19(2):103‐108.

[35] Holyoke P , Oikonen K , Rizzi K , Sethi P (2016). The reflection room: Shifting from deathavoiding to death‐discussing.

[36] Glaser B,G , Strauss AL . Time for dying. Aldine Transaction; 1980.

[37] Schut MS . The dual process model of coping with bereavement: rationale and description. Death Stud. 1999;23(3):197‐224. 10848151 10.1080/074811899201046

[38] MacArtney JI , Broom A , Kirby E , Good P , Wootton J . The liminal and the parallax: living and dying at the end of life. Qual Health Res. 2017;27(5):623‐633. 26658234 10.1177/1049732315618938

[39] Zimbardo P , Boyd J . The time paradox: the new psychology of time that will change your life. Simon and Schuster; 2008.

[40] Sellen K , McGovern M , MacGregor E , Halleran L , Ly L . Concepts of time for HCI research on end of life: exhibit‐based research for: Worksho p HCI at End of Life and Beyond: Proceedings of the International Conference on Human Factors in Computing Systems (VIRTUAL), 27 April–2 May, 2020, CHI '20. ACM, New York, NY; 2020.

[41] Sellen K , MacGregor E , McGovern M , Albess M , Beaubien M , Jang K . Ethical considerations for HCI research on end of life: time moving for: Workshop HCI at End of life and beyond. Proceedings of the International Conference on Human Factors in Computing Systems (VIRTUAL), 27 April 27–2 May, 2020, CHI '20, ACM, New York, NY; 2020.

[42] Sellen K , McGovern M , MacGregor E , Halleran L , Ly L . Time moving: using the participatory exhibit to explore temporal perceptions around death and dying. In: Christer K, Craig C, Chamberlain P, eds. *Full Proceedings of the 6th International Conference on Design4Health*. Vol 3. Sheffield Hallam University; 2020: pp. 193‐200.

[43] O'Reilly M , de Brún T , O'Donnell CA , et al. Material practices for meaningful engagement: an analysis of participatory learning and action research techniques for data generation and analysis in a health research partnership. Health Expect. 2018;21(1):159‐170. 28841753 10.1111/hex.12598 PMC5750692

[44] Death Café . What is a death café? Accessed April 20, 2021. https://deathcafe.com/what/

[45] Death Over Dinner. Accessed April 20, 2021. https://deathoverdinner.org/#who

[46] Bazeley P . Analysing qualitative data: more than ‘identifying themes’. Malays J Qual Res. 2009;2.2:6‐22.

[47] Jewitt C , Oyama R . Visual meaning: a social semiotic approach Van Leeuwen T , Jewitt C , eds. The Handbook of Visual Analysis. SAGE Publications Ltd.; 2004:134‐156. 10.4135/9780857020062

[48] Lövgren M , Hamberg K , Tishelman C . Clock time and embodied time experienced by patients with inoperable lung cancer. Cancer Nurs. 2010;33(1):55‐63. 10.1097/NCC.0b013e3181b382ae 19926976

[49] Anderson F , Downing GM , Hill J , Casorso L , Lerch N . Palliative performance scale (PPS): a new tool. J Palliat Care. 1996;12(1):5‐11. 8857241

[50] McCormack B , Dewing J , McCance T . Developing person‐centred care: addressing contextual challenges through practice development. Online J Issues Nurs. 2011;16(2):3. 22088152

[51] Wolstenholme D , Ross H , Cobb M , Bowen S . Participatory design facilitates Person Centred Nursing in service improvement with older people: a secondary directed content analysis. J Clin Nurs. 2017;26(9‐10):1217‐1225. 27191790 10.1111/jocn.13385 PMC5413812

[52] McCormack B , McCance TV . Development of a framework for person‐centred nursing. J Adv Nurs. 2006;56(5):472‐479. 17078823 10.1111/j.1365-2648.2006.04042.x

[53] Kubler‐Ross E . On death and dying. JAMA. 1972;221(2):174‐179. 10.1001/jama.221.2.174 5067627

[54] Dewey J . Unity of Science as a Social Problem. University of Chicago Press; 1938.

[55] Cowles KV . Issues in qualitative research on sensitive topics. West J Nurs Res. 1988;10(2):163‐179. 10.1177/019394598801000205 3394318

[56] Oikonen K , Wilkes K . Constellations: Designing participatory engagement and end of life. Situated Actions/Proceedings. Accessed August 14, 2021. http://www.pdc2020.org/wp-content/uploads/2020/06/Constellations-Designing-participatory-engagement-and-end-of-life.pdf

[57] Sellen K , McGovern M , MacGregor E , Halleran L , Ly L . Dying. Using a public event series as research tool to open communication on death and dying. In: Christer K, Craig C, Chamberlain P, eds. *Full Proceedings of the 6th International Conference on Design4Health*. Vol 3. Sheffield Hallam University; 2020: pp 201‐207.

[58] Isenberg S , Oikonen K , Saunders S , Wilkes K . Going Home to Die. Dying. Series. 2021. Accessed August 14, 2021. https://www.dyingseries.ca/going-home-to-die.html

